# Breaking the code of silence: Sexual violence and campus culture at Daniel Ouezzin Coulibaly University, Burkina Faso

**DOI:** 10.3389/fsoc.2025.1652314

**Published:** 2026-01-13

**Authors:** Yacouba Tengueri

**Affiliations:** Department of Sociology and Anthropology, Training and Research Unit in Arts and Humanities, Daniel Ouezzin Coulibaly University, Dédougou, Burkina Faso

**Keywords:** gender-based violence, perpetrators and forms of violence, female students, university safety, Burkina Faso

## Abstract

**Introduction:**

Gray literature on gender-based violence (GBV) in universities shows that female students are the most vulnerable group. In our context, this study examines the forms of GBV and the profiles of perpetrators at Daniel Ouezzin Coulibaly University.

**Methods:**

A mixed-methods approach was adopted. The questionnaire survey involved 300 students, and interview guides were administered to 23 participants (students, lecturers, and administrative staff).

**Results:**

Findings indicate that 92.7% of students are familiar with the concept of GBV. Among female students, 45.86% report physical violence, 44.17% psychological or emotional abuse, and 9.97% cultural violence. Sexual harassment through inappropriate touching is a major concern, affecting 30% of respondents. Perpetrators include students (38.46%), lecturers (27.44%), administrative staff (26.92%), and classmates (7.18%).

**Discussion/conclusion:**

These results highlight the high prevalence of GBV in higher education institutions and the urgent need for targeted interventions, including institutional policies, improved infrastructure, and tailored prevention programmes.

## 1 Introduction

During gender and development tutorial sessions with third-year sociology and anthropology students, each student was invited to anonymously report incidents of violence experienced on campus. Students were asked to specify the gender and role of the perpetrator, as well as the types of violence suffered. The analysis revealed multiple forms of gender-based violence, including rape, sexual touching, threats, humiliation, and physical assault. Perpetrators were primarily students, teachers, and administrative staff. These anonymous reports exposed historically unequal power relationships between men and women (Ndiaye, 2021), marked by structural male domination (Bourdieu, 1998). While these tutorial results were surprising to us as the institution's senior administrator, the phenomenon remains largely unaddressed in intellectual circles (Cardi and Pruvost, 2015). However, this violence has always been a reality for women, who are assaulted because they are women (Ratier, 2009). This situation is not a phenomenon specific to Daniel Ouezzin Coulibaly University; it merely reflects society and is very poorly documented. Indeed, as Ndiaye (2021) emphasizes, the sub-region presents the continent's highest index of male domination and gender inequality. Consistent with this, the study by Owusu-Antwi et al. (2024) reveals that Sub-Saharan Africa (SSA) reports the highest prevalence (25.9%) of sexual assault against females in higher education institutions compared to other WHO regions. This crisis is exacerbated by the extremely high rates of psychological violence (64.9%) and stalking (63.7%) found in SSA university settings, thereby compromising students' mental integrity and academic success.

In the scientific literature, it emerges that at the University of New Brunswick, 29.1% of respondents were victims of GBV (Savoie et al., 2018) compared to 36.9% of cases in six (6) Quebec universities (Bergeron et al., 2019). This gender-based violence constitutes learning barriers and contributes to the reproduction of inequalities in girls' educational opportunities for success (Boudon, 1984). This violence is exercised in an environment where dominant (administration, teachers) and dominated (students) relationships are highly visible. Hierarchical relations, processes of stigmatization, and dynamics of social exclusion often contribute to rendering this form of violence invisible. Within the higher education context, sexual harassment is frequently framed as a by-product of the personalized and asymmetrical nature of teacher-student interactions. However, as recent studies demonstrate, harassment should rather be understood as a manifestation of power abuse rooted in the organizational structures themselves (Cabras et al., 2022). Such embedded power asymmetries discourage victims from responding assertively and thereby sustain the invisibility of the phenomenon. Furthermore, insufficient support from academic staff, reflecting a dysfunctional pedagogical relationship, has been identified as a factor significantly correlated with exposure to sexual harassment (Palmieri et al., 2023). This type of relationship increases the risks of abuse of power. Furthermore, it is also noted that cohabitation in student residences; integration evenings or weekends and hazing traditions constitute situations that can generate all forms of sexist and sexual violence. In this situation, victims prefer to keep the trauma of violence secret, and others end up committing suicide. In the USA, the suicide rate is 7 per 100,000 students, equating to 1,100 suicides per year, making it the second leading cause of death. The youngest individuals (aged 15 to 24) and those from marginalized groups are the most likely to experience suicidal thoughts. However, boys who contemplate suicide are more likely to die by suicide compared to girls^1^ Indeed, the work of Combès (2022) on doctoral students in France points the finger at teachers as the main perpetrators of violence against female doctoral students. She traces rapes, touching, humiliation, harassment, depression and, in the worst cases, student suicide.

Some victims blame themselves for the violence they endure, thereby relieving the aggressor of moral responsibility. They bear the burden and relieve the aggressor of all moral responsibility. This behavior is described by Halyna and Volodymyr (2021) as “victim blaming”. For other women, this violence seems “normal” as they believe that men cannot remain indifferent to their beauty. Consequently, the harassment they suffer is minimized or even trivialized. Indeed, according to Ratier (2009), this violence against women is linked to the fact that:

(...) women are both subjects of the unconscious and objects of exchange. We could add that they are objects of love, of desire, pulsional objects and as such likely to attract both libido and beyond libido, the drive in all its forms, including the death drive. (p.53)

Furthermore, the work of Savoie et al. (2018) reveals that for some victims, knowing their aggressor or living with them (in the case of couples) cannot be qualified as violence. In addition, some authors such as Maquestiau and In't Zandt (2017) believe this violence against women worsens according to their socioeconomic situation. The data indicate that socio-economic precarity increases women's exposure to gender-based violence. This heightened vulnerability can be interpreted as a manifestation of the structural inequality between the sexes, a deeply ingrained system of masculine domination which naturalizes female subordination and provides fertile ground for violence (Bourdieu, 1998). As theorized by (Bourdieu 1998), this domination is reproduced and legitimized by the *habitus* and social institutions, rendering its mechanisms often invisible to both those who suffer from it and those who exercise it. Building on this, (Héritier 1996) further argued that the “axiomatic of the difference between the sexes” serves as the universal and organizing foundation for any dualistic worldview, underpinning the differential valence of the sexes and legitimizing male domination. Further research underlines that Domestic Violence (DV) is fundamentally gendered and a direct consequence of patriarchal ideologies. Studies conducted within Indian communities specifically highlight the profound influence of partners' patriarchal beliefs on women's experiences of abuse and controlling behavior, reinforcing a system of male domination and female subordination in the family (Satyen et al., 2024). This article aims to identify the forms and perpetrators of violence in university settings. As Bowen et al. (2018) emphasizes, violence prevention requires a characterization of the types of violence and perpetrators in educational settings.

## 2 Research methodology

To capture the complexity of the field context, a pluralistic methodological approach was employed. This section outlines the adopted methodology, detailing the study area, the population characteristics, the data collection methods and tools, as well as the analytical procedures and ethical considerations.

### 2.1 Study area presentation

UDOC was established as the University Center of Dédougou by decree No. 2010-389/MESSRS/SG/UO of 22 October 2010. It was elevated to the University of Dédougou by decree No. 2017/1307/PRES/PM/MINEFID/MESRSI of 30 December 2017, becoming UDOC following decree No. 2024-0729/PRES/PM/MESRSI/MEFP/MSHP of 28 June 2024. UDOC is located in the Boucle du Mouhoun region, specifically in the village of Souri. UDOC is a state public institution of a scientific, cultural and technical nature, responsible for higher education and research. It consists of two Training and Research Units and two Institutes. The institution enrolled 5,172 students, with 73 % of the student body in the Training and Research Unit in Letters and Human Sciences (UFR-LSH). This institution represents a pertinent research site for this study on gender-based violence, not only due to its sizeable female student population but also because of the socio-economic precarity prevalent among its students. Furthermore, the institution's inherent role as a site for socialization and the reproduction of social norms makes it a privileged field for observing the structural mechanisms of masculine domination.

### 2.2 Study population

This study involved all students enrolled at UFR/LSH. To better understand this phenomenon, we also interviewed female students who were victims of GBV, teachers, academic affairs staff, department heads, and the director and deputy director of UFR-LSH.

### 2.3 Research methods

The conduct of this research relied primarily on mixed methods to understand and explain the extent of this phenomenon in the university environment. Consequently, we combined quantitative and qualitative methods to understand gender-based violence. The mixed-methods approach was selected to enable a comprehensive analysis of gender-based violence within the university. The quantitative component served to establish the prevalence, types and general patterns of violence. Concurrently, the qualitative component was designed to understand the contexts, lived experiences and underlying mechanisms from the participants' perspective. As Hunt and Lavoie (2011) emphasize, the two methods can coexist well because their use allows for counteracting their different limitations.

### 2.4 Sampling and sample

The questionnaire survey employed simple random probabilistic sampling through an online platform (Kobotoolbox). We distributed the questionnaire link via WhatsApp groups to different student cohorts, allowing a 1-month response period. Participants could only complete the online questionnaire once. In total, we obtained 307 participants with 7 invalid forms (unfilled forms and half-completed forms), representing 2.28% of the sample. However, this online survey may contain selection biases (Caumont, 2016) and may not be generalisable (Ghomari, 2022) to all students in the institution. As for qualitative sampling, we adopted a dual technique to understand the phenomenon, namely purposive sampling and snowball sampling in administering our interview guides. We favored the snowball technique given the sensitivity of issues of rape, sexual harassment or sexual touching to identify victims. These are subjects that fall within the intimate, private sphere, sensitive terrains (Tengueri, 2020) and “slippery” ones (Duval, 1987). The final sample comprised 323 participants, with a summary of both the quantitative and qualitative samples provided in the Table 1.

**Table 1:** Sample.

**Characteristics**	**Numbers**
**Quantitative sample**	**300**
Male students	118
Female students	182
**Qualitative sample**	**23**
Director	1
Deputy director	1
Department head	1
Academic affairs officer	1
Female student victims of violence	19

Source: field survey, 2023. Bold represent the size of the surveyed sample.

### 2.5 Data collection tools and techniques

For the questionnaire survey, the Kobo tool box platform was used to design the questionnaire and share it in the different student WhatsApp groups. We disabled information on phone numbers and email addresses of respondents. This decision aimed to ensure participants' anonymity in the survey. The questionnaire was structured around individual variables, knowledge, characteristics and perpetrators of violence in university settings. For the interview guide survey, the semi-structured individual interview technique was used with female students who were victims of sexual violence, but also with teachers and administrative staff. It gives the respondent the opportunity to express themselves freely on aspects of the subject that seem relevant to them at the time of their reflection (Albarello, 1999). The semi-structured interview serves two purposes: to understand the victim's experiences and to create a safe space for disclosure. These emotion-filled moments require the researcher to show empathy toward the victim to encourage them to continue their story. The researcher must work to dispel fear so that the victim tells them their secret. This “symbolic realism” is described by De Sardan (2003) as credit given to the story told by the person surveyed.

### 2.6 Data analysis

For management respecting ethical considerations, we considered confidentiality, anonymity and proper data storage. Therefore, our priority was to respect them and take them into account throughout our work. To this end, each participant was subjected to a consent form. After manually processing the qualitative data, we proceeded with a thematic analysis. Indeed, the analysis of qualitative data was conducted according to the method of (Miles and Huberman 1994), structured around three stages: data reduction through thematic coding, presentation in analytical matrices, and drawing verifiable conclusions. We also relied on the rigorous thematic analysis guidelines developed by Braun and Clarke (2006) to ensure the analytical framework's rigor and traceability. As for the quantitative data extracted in Xls Form file on the Kobo tool box platform and imported into SPSS.20 software to generate statistical tables.

## 3 Results

This section presents the empirical data relating to the individual characteristics of the respondents, their understanding of the concept of gender-based violence (GBV), the various forms of violence experienced within the university setting, as well as the identification of the perpetrators involved on campus.

### 3.1 Sociodemographic characteristics of respondents

Table 1 indicates that the students who participated in this survey comprised were 60.7% female students and 39.3% male students. 12.7% are married, 86% single and 1.3% cohabiting. Furthermore, the vast majority, 93.33%, are aged between [20–30 years], 5.33% between [30–40 years], and 1.34% between [40–50 years]. Moreover, 30% of sexual violence victims were single females. They are aged between 20 and 30 years.

Table 2 indicates the fields and level of study of participants. It shows that 58% of respondents are enrolled in the sociology and anthropology department vs. 42% in the modern letters department. 43.34% were in Bachelor year 3, 29.33% were in Bachelor year 2 and 27.33% in Bachelor year 1.

**Table 2:** Age, sex and marital status of respondents.

**Individual variables**	**Number (*n* = 150)**	**Percentage %**
**Age**		
[20–30]	280	93.33
[30–40]	16	5.33
[40–50]	4	1.34
**Sex**		
Male	118	39.3
Female	182	60.7
**Marital status**		
Married	38	12.7
Single	258	86
Cohabiting	4	1.3

Source: field survey, October 2023.

### 3.2 Students' knowledge of GBV

Gender-based violence was defined differently by our respondents. However, it is noted that they have general knowledge of the concept. For the vast majority (92.7%), it refers to violence against women. Only 7.3% define it as violence exercised by an individual or group of individuals on one or a group of people regardless of gender. None of the respondents defined GBV as violence done solely to men. This is the case of respondent 20, female, aged 28, for whom “GBV is all the violence done to women”.

Respondent 28, aged 24, female, single and enrolled in Modern Letters Bachelor 2: “It is violence exercised on someone without their consent”.

For these respondents, violence can be physical, psychological, moral, economic and cultural. These data reveal that the target audience of this study has knowledge of gender-based violence.

### 3.3 Different forms of GBV encountered within the institution

Table 3 below indicates that students regardless of gender suffer gender-based violence. Physical violence represents 45.86%, psychological violence 44.17% and cultural violence 9.97%. However, cultural violence is suffered outside the university. The types of physical violence identified by respondents include pushing (47.37%), assault and battery (10.53%), sexual violence 30%. Only 12.1% of respondents did not specify the type of physical violence they suffered at UFR-LSH. Respondent 10, single female student in SA1 aged 22: “I was a victim of physical violence at the University Restaurant by a student. A student I don't know came to join the queue. He literally threw me to the ground by pushing me”. As shown in Table 4, the most frequently reported forms of psychological violence are humiliation (19.23%), insults (18.34%), harassment (17.86%), threats (16.57%), discrimination (10.95%), intimidation (10.65%), and blackmail (5.33%). Only 2.07% of respondents did not specify the type of psychological violence they had experienced.

**Table 3:** Field of study and level of respondents.

**Modalities**	**Numbers**	**Percentage %**
**Field of study**		
Sociology-anthropology	174	58
Modern letters	126	42
**Level of study**		
Bachelor 1	82	27.33
Bachelor 2	88	29.33
Bachelor 3	130	43.34

Source: field survey, October 2023.

**Table 4:** Characteristics of types of GBV.

**Type of violence**	**Type of physical violence**	**Percentage**	**Global percentage**
Physical violence	Pushing	47.37	**45.86**
	Assault and battery	10.53	
	Sexual violence	30	
	Not characterized	12.1	
Psychological violence	Humiliation	19.23	**44.17**
	Insults	18.34	
	Harassment	16.86	
	Threats	16.57	
	Discrimination	10.95	
	Intimidation	10.65	
	Blackmail	5.33	
	No response	2.07	
Cultural violence	Early and forced marriage, Levirate, Sororate, Excision	9.97	**9.97**

Source: field survey, October 2023. Bold indicate the overall percentages for each category of violence experienced by the female students.

The types of psychological violence generally suffered are humiliation (19.23%), insults (18.34%), harassment (17.86%), threats (16.57%), discrimination (10.95%), intimidation (10.65%), blackmail (5.33%). Only 2.07% did not characterize the type of psychological violence they suffer.

Testimony 1 (male, 22 years, single): “We, in any case, our teachers spend all their time humiliating us, insulting us like children. When you approach them for information or to understand the course, your heart beats rapidly. They insult you. Especially (....), that's his job. How can a teacher say that his learner is worthless? Me, in any case, when he teaches his class, I go home.” (respondent 17, student in LM, Bachelor 3, single).

Testimony 2 (Female, 21 years, single): “aaah, the administrative staff are extremely difficult to work with. During registration, we encounter problems with the platform. When you approach them, they won't guide you well but they will insult you. They are not welcoming. It's not because we are students that they should humiliate us.”

These statements reveal cases of humiliation, insults, and fear maintained in the relationships between certain teachers and learners. The insults uttered reinforce social distancing and power relations between these different actors in interaction.

To the question “Have you ever experienced a situation of sexual violence?” distinguishing forced sexual touching, attempted forced sexual intercourse and forced sexual intercourse, only 6.12% of female students affirmed having suffered this type of violence in the university environment, yet 30% of respondents had characterized this type of violence. The paradox is that individual interviews with female students reveal cases of attempted rape and non-consensual sexual touching (breasts, buttocks and genitals). This situation characterizes sensitive terrains or subjects. This is what emerges from the testimonies below:

Testimony 3 (female, 25 years, single, third year): “Me, I almost got raped by a teacher. He asked me to bring him some documents. Once in the living room, he threw himself on me. We fought, he tore my dress and injured my hand. I was able to escape from his house. I was very disappointed by his behavior. I was afraid to report him because he was my internship supervisor.”

Testimony 4 (female, 23 years, single, third year): “After class, the teacher asked me for a favor. He told me he wanted to eat tô^2^. For me, it was really tô he wanted to eat. Out of respect for the teacher's authority, I thought it was good to make him tô. When I arrived in front of his hotel, he asked me to come up. Once in his room, he asks me to sit down, I told him it wouldn't be possible because I came with my little sister. He pushed me onto his bed, I got up to flee the room. I didn't go back to get the dish.”

Testimony 5 (female, 20 years, single, first year): “I was raped by a classmate. We were supposed to work on exercises together. Since we see each other every day, I had no reason to think he wanted a romantic relationship with me. As it was about to rain, we went into the living room. That's when he started touching my private parts, I pushed him away. It was like he was possessed, I was afraid he would hurt me. I went home that day sad and dirty in the rain. I told myself it was me who asked for it. If I hadn't gone there.” (respondent 11, first-year student, single).

The testimonies collected expose a multitude of forms of aggression experienced by female students in the university environment. The aggressors are not strangers but known actors with whom they maintain vertical relationships (teachers) or horizontal ones (between classmates). Teachers take advantage of their status as supervisors, authority figures to isolate victims in a private setting. These attempts at rape or sexual touching occur in a context of victim isolation using deception. Furthermore, these statements reveal a blaming of victims who accuse themselves of being solely responsible, thus reinforcing impunity within the university institution.

### 3.4 Identification of perpetrators and prevalence of GBV

Table 5 highlights that students represent 38.46% of cases of violence against female students. Teachers (27.44%), administrative staff (26.92%) and female students' boyfriends (7.18%) are perpetrators cited in cases of violence against female students.

**Table 5:** Perpetrators of violence against female students.

**Perpetrators**	**Numbers**	**Percentage**
Students	70	38.46
Administrative staff	49	26.92
Teachers	50	27.44
Boyfriends	13	7.18
Total	182	100.00

Source: field survey, October 2023.

**Figure 1:**
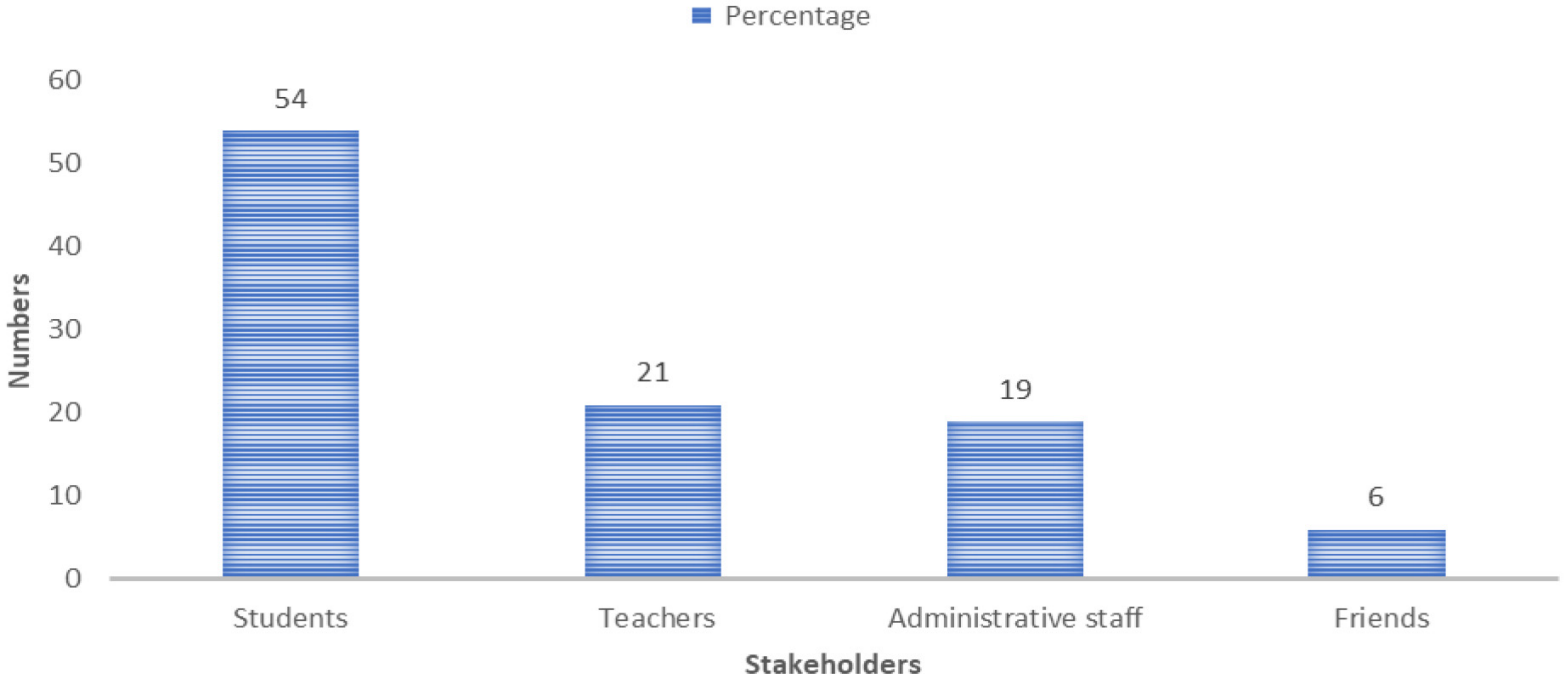
Prevalence of violence. Source: field survey, October 2023.

In terms of prevalence, Figure 1 shows that male students are the most inclined to perpetrate violence, accounting for 54% of reported cases. Lecturers rank second, with a prevalence of 21%, while administrative staff represent 19%. These figures suggest that the majority of incidents originate within the student body, although staff members also contribute significantly to the overall pattern of gender-based violence.

## 4 Discussion of results

The sociodemographic results highlight that the sample is composed predominantly of female students (60.7%), young and single. Multiple forms of violence are distinguished (Raban, 2017) such as physical (jostling, assault and battery, sexual violence), moral or psychological (insult, humiliation, intimidation, etc.) and cultural. However, all victims of sexual violence (sexual touching, rape) are female students, which suggests that the phenomenon affects women more than men. This strong representation of women in the sample could explain the definition of GBV as violence committed primarily against female students (Crenshaw, 1991; Heise, 1998). These results reinforce the notion that GBV is principally directed against women. Like Andoulsi (2022), our respondents define violence as a force exercised upon another person deemed weak and against their consent. This violence can affect their physical, moral, or psychological integrity. For these authors, the absence of consent is the fundamental element in the characterization of sexual harassment.

However, the prevalence of violence at UDOC is substantially higher than rates found in North American universities. Indeed, this rate stands at 29.1% at the University of New Brunswick (Savoie et al., 2018) and 36.9% across six Quebec universities (Bergeron et al., 2019). Nevertheless, our results align more closely with studies conducted in sub-Saharan Africa. In Ghana, Norman et al. (2013) found that 61% of women in medical schools experienced sexual harassment, with perpetrators typically being persons in positions of power; a result comparable to our finding that 27.44% of perpetrators were teachers. Similarly, Mzilangwe et al. (2025) reported across multiple sub-Saharan African countries that power imbalances were identified as the principal risk factor for GBV in universities, with lecturers using their power and influence to harass female students, a trend we also observed at UDOC. Moreover, respondents' youth (aged 20–30 years) and marital status (86% unmarried) make them particularly vulnerable to perpetrators operating within an environment characterized by hierarchical power relations (Kelly, 1988; Walby, 2004). These results align with the conclusion of Bergeron et al. (2019) study, which found that first-year students experience considerably higher rates of exposure to sexual violence (24.7%). However, the socioeconomic dimension appears more pronounced in our context, supporting (Maquestiau and In't Zandt, 2017) argument that poverty amplifies vulnerability to GBV.

Within this hierarchical relationship, masculine domination is most visible (Bourdieu, 1998; Hirata and Kergoat, 2008). According to authors such as Savoie et al. (2018), the university environment is not exempt from these forms of violence in relationships between students and administrative staff. Indeed, these authors observe that 29.1% of respondents at the University of New Brunswick experienced at least one of two forms of sexual violence, namely sexual assault and sexual coercion. As for Bergeron et al. (2019), it emerges that 36.9% of respondents, regardless of sex, affirm having experienced at least one situation of sexual harassment, with 24.7% during their first year at six Quebec universities. These research findings highlight the vulnerability of students enrolled in their first year at university.

Furthermore, the under-reporting of cases of sexual violence; specifically, 6.12% of admitted cases out of 30% of mentioned cases; and semi-structured interviews with students suggest a “culture of silence” (Campbell, 2008; Yoshida and Shanouda, 2015) and “institutional betrayal” Smith and Freyd, (2014) as documented in research on university responses to sexual violence. Our results suggest that fear of being stigmatized or suffering reprisals (Goffman, 1963) from the administration or teachers constrains these victims to engage in self-blame or ‘victim blaming' (Halyna and Volodymyr, 2021) rather than report their aggressors. According to Smith and Freyd (2014)) institutional betrayal' is a transcultural phenomenon. Indeed, in our context, this “culture of silence” is linked to the perception of a raped woman. Marriage being a social privilege, she prefers to remain silent about her rape so as not to reduce her marriage prospects for fear of being humiliated and rejected by her spouse or society. Consequently, they retreat into silence to preserve their dignity. Continuing in this vein, our experience during supervised work also reveals the absence of an institutional reporting mechanism or support service for victims of violence and psychological care without judgement and preconceptions at the time of this study within UDOC. This complete absence of abuse and violence reporting services is often compounded by the trivialization and normalization of non-physical aggression (such as verbal or sexual abuse) against women. Specifically, within academia, organizational and institutional factors play a key role in the experience of reporting sexual harassment (SH). There is an urgent need for academic institutions to create accessible procedures to facilitate SH reporting and provide robust support to victims (Smith and Freyd, 2014; Arya and George, 2022; Cabras et al., 2022). The authors describe this situation in their work as “institutional betrayal”. Consequently, victims often choose to remain silent to preserve their dignity, a situation exacerbated by the observed absence of dedicated institutional mechanisms for listening and providing non-judgemental psychological support. This deficiency corresponds to the concept of institutional betrayal as defined by (Smith and Freyd 2014). The university thus appears as a space for the reproduction of sexual inequalities, marked by culpable tolerance and structural complicity (Combès, 2022) toward the violence suffered by the entire university community. This dynamic is reinforced by local representations that trivialize abuse, where notions such as “PAM” (“Petits Avantages du Métier” or “Minor Professional Perks”) or the “common fund”, even when mentioned jokingly, legitimize predatory power relations. Sexual predation is thus perceived as an advantage linked to hierarchical status, which contributes to normalizing vertical violence within the institution.

From this study, it emerges that the identity of perpetrators further complicates the reporting of the phenomenon at the university. Indeed, administrative staff and teachers constitute approximately 54.36% of the perpetrators in different forms of violence within the university space, which reflects a diversity of forms of abuse of power. Administrative staff and teachers are holders of power and consequently possess the means to abuse this power relationship. They can, in cases of refusal or reporting, compromise victims' studies or make their success conditional. These results concerning the perpetrators of violence align with the work of Combès (2022) in France, where doctoral students appear as prey to their supervisors. There is consistency in the identification of perpetrators which allows us to affirm that the abuse of hierarchical power within higher education institutions facilitates GBV, regardless of geographical and institutional space. However, this violence is not exclusive to those who maintain this type of vertical relationship (authority) with students; it also appears that 38.46% of perpetrators have a horizontal relationship (between peers) with victims. This result differs from certain Western studies whose conclusions focus more on teachers' professional misconduct (Cantalupo and Kidder, 2018; Wiley and Young, 2021). In this type of horizontal relationship, one would then speak of ‘toxic masculinity' (Grenier, 2020) amongst students who perpetrate violence toward their peers. These acts of violence are not without consequences for the psychological stability and learning capacity of violence victims (Van Der Kolk, 2014). This is apparent in the semi-structured interviews conducted with victims. These victims reveal anxiety and guilt in their discourse. These post-traumatic syndromes of rape victims are extensively documented by (Herman 1992) in this work. The psychological consequences of GBV are complex. Beyond acute trauma, the process of separating from an abusive partner is recognized as a multifactorial, non-linear, and dynamic process, often governed by a complex interplay of forces that either facilitate or accelerate the separation. Furthermore, research directly links gender-based violence exposure to severe psychological distress in female university students, manifesting as Post-Traumatic Stress Disorder (PTSD), depression, and anxiety. This impact directly contributes to victims risking being emotionally affected and having their learning capacity affected (Di Basilio et al., 2022; Laz-Figueroa et al., 2025).

While Western universities have established GBV policies, UDOC lacked formal frameworks during this study. Title IX mandates comprehensive procedures at U.S. federally funded institutions (U.S. Department of Justice, 2015), and European universities demonstrate institutional infrastructure, though unevenly implemented across 46 institutions in 15 countries (Lipinsky et al., 2022). Sub-Saharan African universities exhibit fragmented policy frameworks (Olawale et al., 2024), with UDOC's complete absence representing severe structural deficits. Beyond policy differences, GBV manifestations vary geographically. Western research predominantly examines sexual harassment and assault, with 31.5% of American undergraduate women experiencing non-consensual contact (Cantor et al., 2020) and 62% of European students reporting GBV (Lipinsky et al., 2022). This study uniquely identified cultural violence (9.97%), including forced marriage and female genital mutilation, highlighting West African context-specific dimensions. Despite variations, patriarchal hierarchies within universities globally create environments where violence maintains male dominance (Kaufman, 2023), demonstrating both universal and context-specific dimensions of campus-based GBV.

## 5 Conclusion

The main objective of this article was to identify the types and perpetrators of violence against students at UDOC. The mixed approach adopted highlights physical, psychological and cultural violence. While all students are victims of violence, female students suffer more sexual violence within the institution. Gender, age, marital status and level of study are common vulnerability factors for violence victims. Generally, female student victims suffer harassment, sexual violence, insults, humiliation, assault and battery, etc. Furthermore, the typology of aggressors (administrative staff, teachers in majority) reflects an abuse of power within the institution. These results explain this “culture of silence” in reporting the phenomenon within the institution. Furthermore, this “culture of silence” originates in the absence of a reporting mechanism, but also the fear of being stigmatized and rejected by society. Consequently, victims' risk being emotionally affected and having their learning capacity affected. To mitigate the impact of GBV on student life, UDOC must undertake three complementary actions: the adoption of regulatory frameworks, the raising of awareness across the university community, and the deployment of dedicated services for reporting abuse and providing psychological support to victims. The recommended deployment of dedicated services for psychological support must be informed by research on revictimisation, which should be viewed as a dynamic process involving multiple forces. Interventions should thus prioritize victims' emotional resources, affect regulation skills, and social support networks, especially for women with cumulative victimization histories. Concurrently, UDOC should consider preventive approaches targeting perpetrators. For instance, the “Environmental Corrections” framework suggests supervision strategies should focus on reducing opportunities for reoffending (opportunity-reduction strategies), rather than relying exclusively on generic deterrence tactics (Bellot et al., 2022; Schaefer et al., 2022).

## Data Availability

The datasets presented in this article are not readily available because no data restrictions. Requests to access the datasets should be directed to yacouba.tengueri@univ-dedougou.bf.

## Footnotes

Reviewed by: Aulia Nursyifa, Pamulang University, Indonesia Blessing Ngowari Ramsey-Soroghaye, University of Port Harcourt, Nigeria

## Footnotes

1 BrainsWay (2025). College student suicide: Failures and potential solutions. BrainsWay. https://www.brainsway.com/knowledge-center/college-student-suicide-failures-and-potential-solutions/.
